# Water Dynamics in *Shewanella oneidensis* at Ambient and High Pressure using Quasi-Elastic Neutron Scattering

**DOI:** 10.1038/srep18862

**Published:** 2016-01-07

**Authors:** Fabrizia Foglia, Rachael Hazael, Giovanna G. Simeoni, Marie-Sousai Appavou, Martine Moulin, Michael Haertlein, V. Trevor Forsyth, Tilo Seydel, Isabelle Daniel, Filip Meersman, Paul F. McMillan

**Affiliations:** 1 Chemistry Department, Christopher Ingold Laboratories, University College London, 20 Gordon Street, London WC1H 0AJ, UK; 2 Heinz Maier-Leibnitz Zentrum (MLZ) and Physics Department, Technisches Universität München, Lichtenbergstrasse 1, D-85748 Garching, Germany; 3 Jülich Center for Neutron Sciences at MLZ, Forschungszentrum Jülich GmbH, Lichtenbergstrasse 1, D-85748 Garching, Germany; 4 Life Sciences Group, Carl-Ivar Brändén Building, Institut Laue-Langevin, 71 avenue des Martyrs, 38042 Grenoble cedex 9, France; 5 Faculty of Natural Sciences/ISTM, Keele University, Staffordshire ST5 5BG, UK; 6 Science Division, Institut Laue-Langevin, CS 20156, 71 avenue des Martyrs, 38042 Grenoble cedex 9, France; 7 Laboratoire de Géologie de Lyon, UMR 5276, Université Lyon 1-ENS de Lyon-CNRS, 2 rue Raphaël Dubois, 69622 Villeurbanne, France; 8 Biomolecular & Analytical Mass Spectrometry, Department of Chemistry, University of Antwerp, Groenenborgerlaan 171, B-2020 Antwerp, Belgium; 9 Department of Chemical Engineering, Imperial College London, London SW7 2AZ, UK

## Abstract

Quasielastic neutron scattering (QENS) is an ideal technique for studying water transport and relaxation dynamics at pico- to nanosecond timescales and at length scales relevant to cellular dimensions. Studies of high pressure dynamic effects in live organisms are needed to understand Earth’s deep biosphere and biotechnology applications. Here we applied QENS to study water transport in *Shewanella oneidensis* at ambient (0.1 MPa) and high (200 MPa) pressure using H/D isotopic contrast experiments for normal and perdeuterated bacteria and buffer solutions to distinguish intracellular and transmembrane processes. The results indicate that intracellular water dynamics are comparable with bulk diffusion rates in aqueous fluids at ambient conditions but a significant reduction occurs in high pressure mobility. We interpret this as due to enhanced interactions with macromolecules in the nanoconfined environment. Overall diffusion rates across the cell envelope also occur at similar rates but unexpected narrowing of the QENS signal appears between momentum transfer values *Q* = 0.7–1.1 Å^−1^ corresponding to real space dimensions of 6–9 Å. The relaxation time increase can be explained by correlated dynamics of molecules passing through Aquaporin water transport complexes located within the inner or outer membrane structures.

Water dynamics inside cells and across the cell envelope are key to biological processes^1^,^2^,^3^,^4^. Although it is thought that water mobility must be significantly slowed by macromolecular interactions within the crowded intracellular environment, results from NMR and quasi-elastic neutron scattering (QENS) studies have revealed diffusion rates that more closely resemble bulk aqueous solutions^5^,^6^. QENS is a powerful technique for studying correlated proton dynamics and relaxation processes over a wide range of momentum (*Q*) and energy (ħω) transfers at nano- to picosecond timescales relevant to cellular biology^7^,^8^,^9^. In our experiment these correspond to a spectral broadening of the elastic line exceeding the energy resolution by a few tenths of meV. Therefore, the dynamics probed by QENS are readily distinguished from vibrational excitations or events such as enzyme catalysis that occur on significantly faster (fs) or slower (ms) timescales, respectively. QENS signals arise from incoherent scattering processes. Because of the large differences in H/D neutron scattering cross sections, isotopic contrast experiments can be designed to focus on the dynamics occurring within specific regions of the biological cell, including the intracellular medium (Im) or across membrane structures. QENS has already been developed to study high pressure effects on dynamic processes both in aqueous media and for biologically important macromolecules^10^,^11^. Here we extended the application of the QENS technique to investigate water dynamics in H/D substituted samples of live *Shewanella oneidensis* bacteria at ambient (0.1 MPa) and high (200 MPa) pressures relevant to subsurface biological processes.

Observations of live bacteria at >10 km below the ocean surface as well as samples recovered from continental and suboceanic drilling projects have revealed microbial lifeforms existing at up to at least P = 110 MPa^12^,^13^. Understanding the behavior of microorganisms exposed to HP conditions is also necessary to optimize food preservation and other biotechnologies where pressures in the 200–700 MPa range are applied to eliminate harmful bacteria^14^. We selected *S. oneidensis* as a model for our study. This prokaryotic organism exists under both aerobic and anaerobic conditions and its genus contains several piezophilic (“pressure-loving”) species^15^,^16^,^17^. Its metal reduction (MR) capacities are being harnessed for environmental remediation and fuel cell applications^18^,^19^, and its metabolic processes have been studied by *in situ* spectroscopy under HP conditions^20^,^21^,^22^. *S. oneidensis* has also been investigated to study its pressure adaptation and survival under conditions extending into the GigaPascal (P > 1 GPa) range^20^,^23^.

## Results and Discussion

**Figure 1:**
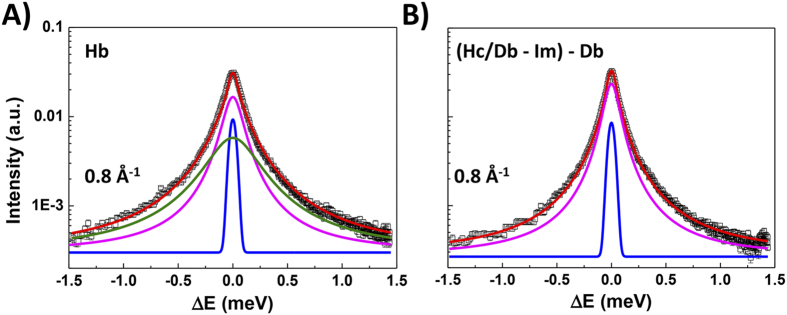
Analysis of the neutron dynamic scattering function S(*Q*, ω) at ambient pressure (0.1 MPa) and *Q* = 0.8 Å^−1^ for the hydrogenated buffer solution ((**A**): Hb) used for bacterial suspensions and an isotopic contrast ((**B**): [Hc/Db-Im]-Db; Im represents the intracellular medium) designed to highlight dynamics across the cell envelope. The data and lineshape fits are shown with a logarithmic intensity scale to highlight QENS contributions. The central Gaussian (blue) due to elastic scattering defines the instrumental resolution function. For aqueous solutions both translational (magenta) and rotational (green) Lorentzian components were used to fit the QENS lineshapes. These two components are related to τ_D_ (translational relaxation component corresponding to water diffusion) and τ_R_ (molecular rotational relaxational processes). The global fit (red continuous curve) is overlain on the data points (black). The isotopic contrast datasets (**B**) used to analyse transmembrane dynamics were fit using a single Lorentzian function. The energy transfer scale shown in meV is converted to reciprocal time (s^−1^) units using ΔE = ħω.

Our QENS experiments were carried out at the time-of-flight spectrometer TOFTOF (Garching, Germany)^24^ that allowed recording with low background levels necessary for successful analysis of the H/D isotopic contrast experiments, over a wide *Q* range (0.2–1.8 Å^−1^) corresponding to real space correlation lengths ~3–30 Å (Fig. 1). A detailed discussion of the experimental details, including sample preparation, instrumentation and data analysis protocols is presented given in the Materials and Methods section below.

### Bulk aqueous buffer solutions

**Figure 2:**
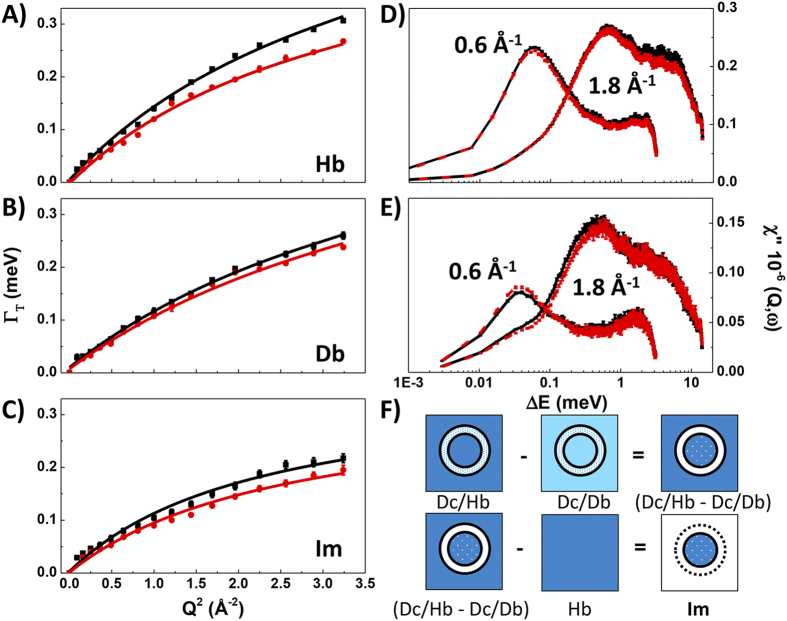
Water dynamics in the aqueous suspension (panel (**A**): Hb, H-buffer; panel (**B**): Db, D-buffer) and intracellular medium (panel (**C**): Im) from analysis of the QENS linewidth (Γ for the translational diffusion component) as a function of momentum transfer (*Q*^2^). The isotopic contrast combination used to obtain Im was [(Dc/Hb–Dc/Db)–Hb] (panel (**F**)). At left are shown the Γ_T_(*Q*^2^) dependencies at 0.1 (black) and 200MPa (red) fit to a *SS* jump model. At right are shown the χ′(*Q*, ω) susceptibility functions determined at *Q* = 0.6 and 1.8 Å^−1^ for the aqueous solutions.

We tested our approach by examining water dynamics in the bulk aqueous buffer solutions (non isotopically substituted buffer Hb; perdeuterated (98%) buffer Db) that served as the bacterial growth and suspension media for our experiments. The main electrolyte species present are Na^+^ and Cl^−^ with structure-breaking to borderline properties along with structure-making phosphate anions^25^. The QENS broadening arising from diffusive mechanisms can be described by a sum of Lorentzian functions, whose number and relative contribution depends on the specific processes involved. Focusing on the main narrow component associated with translational relaxation, fitting the full width at half maximum (FWHM) Γ_T_(Q^2^) relations to a Singwi-Sjölander (*SS*) jump model^7^ (see Methods section below) led to diffusion coefficients D_T_ = 2.70_±0.20_ × 10^−5^ and 2.00_±0.11_ × 10^−5^ cm^2^s^−1^ for Hb and Db, respectively, at ambient conditions (Fig. 2A,B, Table 1). These results are in excellent agreement with tabulated values for water and aqueous solutions at or near ambient temperature. At 200 MPa the diffusion coefficients are lowered by 9–11% for the aqueous electrolyte solutions. The reduced QENS linewidths observed for Db *vs* Hb liquids at both pressures occur due to de Gennes narrowing, combined with atomic mass effects on the molecular diffusion coefficients^7^,^11^,^26^,^27^.

As we investigated the Γ_T_(Q^2^) properties of each dataset, we also carried out a complementary examination of the susceptibility function χ′(*Q*, ω) normalized to the frequency (energy transfer) scale^28^,^29^. At finite temperature the intensity of any experimentally measured scattering function S(*Q*, ω) is asymmetric with respect to the energy loss (ω) axis due to the Bose-Einstein thermal population factor. Studying the form of χ′(*Q*, ω) reveals the proper distribution of spectral weight among the different dynamic processes, along with additional useful information concerning the static structure factor S(*Q*). Furthermore, using a stretched energy scale with respect to the corresponding S(*Q* = const, ω) spectrum allows a clear separation between and determination of the number of relaxation mechanisms contained within the density of states responsible for the diffusive dynamics. Similar interpretations can also be achieved by developing a sophisticated analysis of relationships between fitting parameters used to reproduce the QENS linewidth result; however, we preferred to crosscheck the fit analyses by using the χ′(*Q*, ω) results as an independent method. For simplicity we show the details only for the case of the bulk aqueous solutions, even though its analysis has supported the interpretation of all the reported results and also provided an *in situ* marker of the hydrostatic conditions achieved during the experiments. In Fig. 2D,E we report the imaginary susceptibility of both Hb and Db at two *Q* values representative of long (*Q* = 0.6 Å^−1^) and short (*Q* = 1.8 Å^−1^) range dynamics. In the case of the Hb data (Fig. 2D), corresponding to a mostly incoherent signal, the two observed peaks have comparable intensity. However, in the case of Db (Fig. 2E), where the signal is dominated by coherent scattering, the intensity of the peaks is considerably modified by the influence of the static structure factor S(*Q*).

### Intracellular medium (Im)

**Figure 3:**
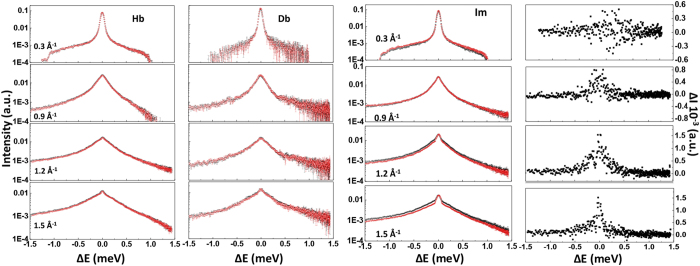
Selected QENS data at different *Q* values and 0.1 *vs* 200 MPa pressures. Left panel: Comparison between neutron dynamic scattering functions S(*Q*, ω) at 0.1 (black symbols) and 200 MPa (red symbols) for aqueous buffer solutions (H-buffer, Hb and D-buffer, Db) at *Q* = 0.3, 0.9, 1.2 and 1.5 Å^−1^. Right panel: Comparison between neutron dynamic scattering functions S(*Q*, ω) at 0.1 (black symbols) and 200 MPa (red symbols) for water dynamics within the intracellular medium (Im, obtained from the following subtraction between isotopic contrasts: [(Dc/Hb–Dc/Db)–Hb]) at *Q* = 0.3, 0.9, 1.2 and 1.5 Å^−1^, showing the development of a significant pressure effect on the QENS profile at high *Q* values. This is absent in the case of Hb and Db buffer solutions. At far right are shown the corresponding subtractions between Im scattering profiles obtained at 0.1 and 200 MPa to highlight the effect of pressure on the QENS profile.

We next examined water dynamics in the intracellular medium (Im) using isotopic contrasts derived from QENS datasets for: (i) unlabelled bacterial cells (Hc) suspended in deuterated buffer solution (Hc/Db); (ii) perdeuterated cells (Dc) suspended in deuterated buffer (Dc/Db) and (iii) perdeuterated cells in unlabelled buffer (Dc/Hb), to construct the isotopic contrast [(Dc/Hb–Dc/Db)–Hb] (Fig. 2, Table 1). Fitting the Γ_t_(*Q*^2^) data by the SS jump model led to an intracellular diffusion coefficient D_T_ = 2.43_±0.14_ × 10^−5^ cm^2^s^−1^, intermediate between the values for Hb and Db buffer solutions. This result indicates that water diffusion in the intracellular medium is indistinguishable from the bulk over the examined Q-range corresponding to correlated motions extending between approximately 3–30 Å in real space. Jasnin *et al*. likewise observed no differences in *E. coli* cytoplasm dynamics from diffusion in bulk aqueous media examined by QENS at ambient pressure^6^,^7^,^8^,^9^. Similar experiments on hydrogels with water confined within nanoscopic pores show that water diffusion over these length scales is essentially the same as that in bulk fluids^30^. As pressure was increased to 200 MPa we observed that the cytoplasmic mobility in *S. oneidensis* became reduced by 18%, nearly twice that observed for the Hb or Db aqueous buffer solutions (−11%, −9%) (Fig. 2C, Table 1). The pressure effect on the Γ_T_(Q^2^) is already obvious from initial inspection of the QENS data sets that clearly show an enhanced signal at large *Q* values for the Im compared with bulk Hb, Db buffer solutions (Fig. 3). This dynamic slowdown is accompanied by a 26% increase in the rotational relaxation time τ_R_, compared with only 10–18% for the bulk aqueous electrolyte solutions (Table 1). We suggest that these effects indicate enhanced interactions between H_2_O and intracellular macromolecules becoming more dominant as the volume within the cell is reduced at high pressure.

### Transport across the cellular envelope

**Figure 4:**
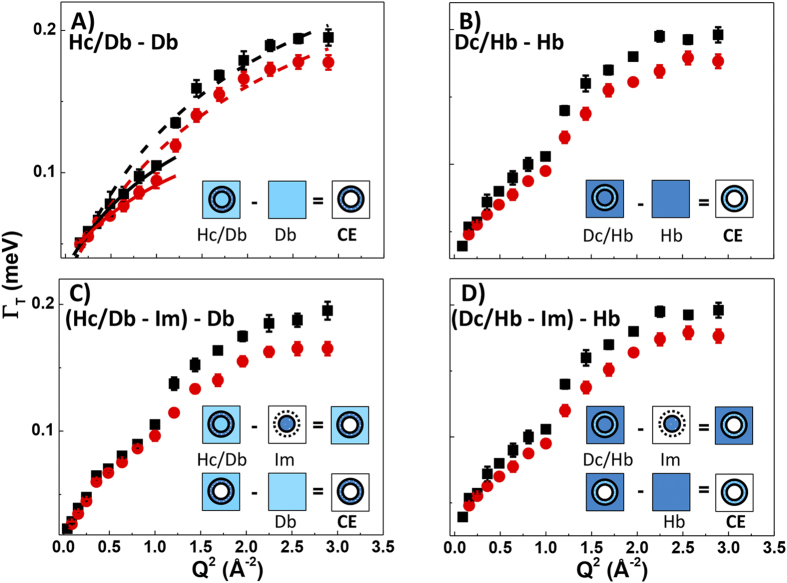
Water dynamics across the cell envelope (CE) at 0.1 (black) and 200MPa (red), visualised using different isotopic contrast subtractions. (**A**) (Hc/Db – Db), with *SS* jump model fits to the overall cell envelope (0.2–1.8 Å^−1^) shown as dashed lines and restricted to the 0.2–1.0 Å^−1^ range (solid lines). (**B**) Data points for the corresponding isotopic contrast (Dc/Hb – Hb). Panels (**C**) and (**D**): isotopic contrasts [(Hc/Db – Im) – Db)] and [(Dc/Hb – Im) – Hb)] showing data points taking into account differential effects of pressure on Im compared with bulk media (Hb, Db).

To investigate water transport across the cell envelope we first examined the isotopic contrasts (Hc/Db–Db) and (Dc/Hb–Hb) assuming that water diffusion rates inside the cell remained the same as in bulk aqueous media at high pressure. In a second refined version of the model we then constructed the isotopic contrasts [((H/D)–Im)–Db] and [((D/H)–Im)–Hb] that take into account the differential effect on Im diffusion at pressure compared with the Hb and Db buffer solutions noted above (Fig. 4). Both approaches gave similar results.

The Γ_T_(*Q*^2^) data present a similar picture of water dynamics to the bulk and Im media but an unexpected narrowing of the QENS linewidths occurs between Q^2^ = 0.5–1.2 Å^−2^. A small downwards deviation in Γ(Q^2^) does appear to be present for the Im data set (Fig. 2) between Q^2^ = 1–2.3 Å^−2^, however, that is much less pronounced than for the isotopic contrast datasets used to analyze the transmembrane behavior. We note that the Im dataset subtractions used to study the intracellular dynamics (Im) might have been slightly affected by transmembrane water diffusion processes, but to a much smaller extent than observed for the datasets presented in Fig. 4 and discussed in this section.

Fitting the SS model to the ensemble of data points at long (*Q* < 0.7 Å^−1^) and short (*Q* > 1.1 Å^−1^) correlation lengths we obtained D_T_ = 2.43–2.64 × 10^−5^ and 1.95–2.10 × 10^−5^ cm^2^s^−1^ (at 0.1 and 200 MPa, respectively), depending on the isotopic contrast model used (dashed line fit illustrated in Fig. 4, panel A). These values represent the overall diffusion coefficients throughout the entire cell envelope. Both they and their pressure dependence are nearly identical to those for diffusion within the Im (Table 1). However, we note that if only data from longer correlation lengths (*Q* < 1 Å^−1^) had been considered in the QENS analysis (solid line in Fig. 4A) the result would indicate significantly lowered apparent transmembrane diffusion coefficients ranging between 1.75–1.82 × 10^−5^ and 1.52–1.72 × 10^−5^ cm^2^s^−1^ (i.e., at 0.1 and 200 MPa, respectively) (Fig. 4, Table 1).

We now examine the implications of these results and provide an initial interpretation of the unusual form of the Γ_T_(*Q*^2^) relations. An initial downwards deviation of the data from the initial progression of the curve near 0.7 Å^−1^ is followed by a rapid upturn as the data regains the “normal” diffusion relation at approximately 1.1 Å^−1^. In an early analysis of the data, we considered the possibility that this might be associated with two superposed motions (i.e., transmembrane diffusion *vs* nanoconfined water dynamics within the membrane structures). However, it quickly became clear that the jump model, or any superposition of jump models, could not describe the water dynamics in any meaningful or justifiable way in terms of likely relaxation or nanoconfinement scenarios. However, a simpler model can be advanced to interpret the transmembrane Γ_T_(*Q*^2^) relations by examining the relaxation timescales implied by the QENS data, along with models that are becoming established for molecular water transport across the cell membrane structures *via* Aquaporin protein complex channels.

First we note that the unexpected slope changes can be interpreted as a “dip” relative to the Γ_T_(*Q*^2^) relation expected for a jump diffusion model applied to a single diffusion process, between *Q* = 0.7–1.1 Å^−1^. Each data point within this range represents an unexpected narrowing of the QENS linewidth and hence a correspondingly enhanced relaxation time for those correlated water transport motions occurring with correlation lengths between 6–9 Å in real space (Fig. 4). This spatial dimension is identified with that of the central region of Aquaporin (Aqp) channels that are already known to transport water molecules across the cell membrane in animal and bacterial cells^31^,^32^,^33^,^34^. AqpZ is known to be present in *Shewanella*. We note that all four isotopic contrast combinations shown in Fig. 4 show the same result. If the anomaly in the Γ_T_(*Q*^2^) datasets had been due to a systematic instrumental artefact, this would have been removed by the subtraction procedures. However, if such an artefact had been present in only one of the datasets, this would have given rise to anomalous slope changes occurring in opposite directions for different contrast combinations. The data in Fig. 4 show that this is clearly not the case, and we can examine the physical interpretation of the QENS line narrowing effect.

MD simulations combined with X-ray crystallography have previously shown that approximately 7–9 H_2_O molecules traverse the Aqp channel in single file in a spatially correlated motion, with a reversal of the molecular orientation occurring midway through the channel that ensures transport *via* intact molecular units^34^. Invoking such highly correlated H_2_O transport dynamics can then explain the unusually long lifetime recorded by our QENS data, that correspond to 6.3 ps at 1 Å^−1^, compared with 5.3 ps expected by interpolating between the bulk diffusion dynamics occurring at longer and shorter *Q* values (Fig. 3). Our QENS isotopic contrast experiments thus provide support for the model of highly correlated water transport dynamics occurring within transmembrane Aqp channels of *S. oneidensis*. The QENS linewidth exhibits a further reduction at 200 MPa indicating an ~7% increase in correlation time for water molecules in the transport channel as the applied pressure is increased (Fig. 4). This observation will have implications for the way in which water is transported across the cell wall for organisms subjected to high pressure conditions.

## Conclusion

We used QENS at ambient (0.1 MPa) and high pressure (200 MPa) combined with a range of H/D contrast experiments on isotopically labelled samples to reveal details of water dynamics occurring within the intracellular medium and across the cell membrane for *Shewanella oneidensis* bacteria suspended in their growth/buffer media. The QENS linewidth data were analyzed according to molecular jump models to examine the effects of pressure on the water transport processes. At ambient pressure the intracellular (Im) mobility is indistinguishable from bulk water dynamics over correlation lengths extending between 3–30 Å. However, the reduction in diffusion coefficients with pressure is significantly greater than for the bulk aqueous solutions indicating the presence of interactions with macromolecules within the nanoconfined cellular environment. The overall water diffusion rate across the cell envelope is similar to that in the intracellular medium at ambient and high pressure. However, an unusual narrowing of the QENS line occurs for *Q* = 0.7–1.1 Å^−1^ corresponding to an enhanced relaxation time for correlated water transport over a real space distance ~6–9 Å. This corresponds to H_2_O molecules passing through the transmembrane Aqp channels embedded within the outer or inner cell membrane structures. Our HP-QENS studies do not reveal remarkable effects on H_2_O diffusion either inside the cells or across the cell membrane at pressures up to 200 MPa, that are relevant to the range in which subsurface microbial life has been shown to exist. *In situ* studies of both aerobic and anaerobic metabolic processes enabled by monitoring the rates of formate oxidation and Se^4+^ or Fe^3+^ reduction have demonstrated a >3-fold decrease in respiration rates within the same pressure range^20^,^21^,^22^. Our QENS results reveal that this metabolic slowdown is not directly coupled to the intracellular or transmembrane water diffusion rates at least on the molecular length and time scales probed here.

## Materials and Methods

### Bacterial sample preparation and isotopic substitutions

*S. oneidensis* samples (CIP 106686) obtained from the Collection Institut Pasteur (Paris, France) were grown at 30 °C in LB broth at 180 rpm after selecting a single colony from a LB agar plate and harvested at stationary phase (10^8^ cells/mL). A perdeuterated strain was grown at the Deuteration Laboratory (ILL-PSB, Grenoble, France). The D-silantes medium used to grow and deuterate the bacteria prior to the neutron scattering experiments led to >98% labeling^35^. Bacterial samples were prepared at a concentration of 50 mg/mL and data recorded with acquisition time 6 h for each pressure point. The pressure ramp used to reach the target value was 20 MPa/min. Complementary laboratory experiments carried out using similar protocols to previous work^23^ showed a 14% bacterial survival rate following pressurization at 200 MPa for 6 h.

Because H or D incorporated within the macromolecular cellular structures are primarily non-labile^36^, our isotope contrast experiments allowed us to focus attention on mobile water species diffusing over correlated length scales up to 30–40Å, that are relevant to intracellular and trans-membrane dimensions. Proton exchange reactions and vibrational excitations within these systems occur on either much slower (ms) or faster (fs) timescales that were not imaged by our QENS experiments that are restricted to the picosecond (ps) regime.

The dataset subtractions made to construct the isotopic contrasts were enabled by the large difference in incoherent scattering cross section between H and D (2.05 and 80.27 barn, respectively), taking account of the fact that D is almost invisible relative to H when both are present. The D-labelled growth/suspension medium was not 100% deuterated and the resulting perdeuterated cells also retained a small quantity of hydrogenated component. Because of the scattering cross sections involved, the isotopic contrast Dc/Db could be used to remove most of the coherent scattering contribution that is mainly present in the elastic line, as well as incoherent scattering from the H-component that is essentially located in the inelastic part of the scattering pattern, so that the results we report are truly QENS data sets. The presence of the isotopic impurity also helped ensure that an incoherent signal was observed in these Dc/Db experiments.

Some D/H exchange occurs naturally *via* exchangeable protons when D-cells are placed in H-buffer, and *vice versa*. Each sample was resuspended three times in the relevant isotopic medium and left to equilibrate for at least 30 minutes before any data acquisition in order to ensure complete re-equilibration among any chemically exchangeable protons and deuterons in the bacterial sample^37^,^38^.

### QENS experiments and data treatment protocols

QENS experiments were carried out at the time-of-flight spectrometer TOFTOF (Garching, Germany)^24^ that allowed recording with low background levels necessary for successful analysis of the H/D isotopic contrast experiments, over a wide (0.2–1.8 Å^−1^) *Q* range. TOFTOF is a direct geometry spectrometer: the *Q* resolution is ensured by the large solid angle covered by the detector area, whereas the energy of the scattered neutrons is measured after the time-of-flight for travelling from the sample to the detectors. The instrumental energy resolution (0.06 meV) defined by the incident neutron wavelength (λ = 6 Å) and chopper frequency (12000 rpm) produced a constant Gaussian lineshape that was perfectly suited to the characterization of the QENS signals of interest.

Our experiments that present a new approach using neutron scattering techniques to investigate important biological and biophysical dynamics required about 18 months of dedicated technical and conceptual development. It is important to evaluate the reproducibility and reliability of our findings. We repeated the experiments twice over a period of several months to allow an assessment of all potential sources of uncertainty ranging from sample growth, deuteration and resuspension protocols, through possible changes in samples occurring during shipping and handling, to instrumental issues associated with neutron flux, humidity accumulation on detectors, cell alignment, etc., and testing of software and protocols for data handling. The two independent datasets provided fully consistent results, and they could be averaged to provide error bars that we associated with each experimental data point. These error bars were so minor that they appear included within the size of the data points shown in Fig. 2, for example. This gives us confidence in the physical interpretation of our results. However, we note that similar experiments should be carried out at other neutron scattering facilities to fully demonstrate the power of this valuable technique to give new information on the dynamics of water and biological macromolecules in live organisms potentially as they are exposed to extreme environmental conditions.

The neutron scattering data were handled and treated using the Large Array Manipulation Program (LAMP)^39^. This software package provided the standard routines for the proper calibration and normalization of TOF data files and converted the matrix I(2Θ, t) into the dynamic structure factor S(*Q*, ω) of interest. Data analysis was performed on spectra at different constant *Q* values. Further details are given below. HP-QENS data were acquired using an Al-body cell with an internal gauge used to continuously monitor the pressure. In contrast to many high pressure investigations that are affected by a huge background from the sample environment, the measurement of a liquid solution in such an Al-cell represents a favorable condition for QENS studies. At an incident wavelength of 6 Å the pressure cell is almost transparent: i.e., it exhibits 97% neutron transmission overall, no incoherent scattering in the QENS region, Bragg peaks outside the accessible *Q*-range, and phonons well separated in energy from any liquid features. Moreover, we chose a scattering geometry with the high pressure cell rotated by 45° clockwise with respect to the propagation axis of the incident neutron beam, thus allowing a continuous signal acquisition over the entire detector area (i.e., with no shaded detector area). Further details concerning the high pressure cell and the scattering geometry are provided in^11^.

Raw data files were normalized to the neutron flux and the acquisition time, thus removing any dependence on the neutron source and the experimental duration. The global detector efficiency was calibrated to the energy of the incoming neutron beam. Further detector-specific efficiency fluctuations were corrected individually after comparison with the purely incoherent signal scattered from an elemental vanadium (V) reference sample. This constituted a 1.1 mm thick flat plate that also provided a calibrant for the experimental elastic resolution function. Indeed to guarantee a uniform normalization over all spectra contained within a workspace dataset, the time-resolved scattered intensity recorded by each detector was normalized to the integrated elastic intensity scattered by the vanadium reference standard at the same 2Θ angular position.We note that in the case of direct geometry TOF spectrometers, the *Q* resolution is provided by the distinct angular coordinate 2Θ assigned to each detector. Time-of-flight and angular units were then converted into the energy and momentum transfer scale according to standard LAMP routines, developed to provide the exact transformation of the double differential cross section into the corresponding dynamic structure factor S(*Q*, ω). The intensity data from scattered neutrons were either grouped according to a regular (*Q*, E) grid (linear binning) to provide information on S(*Q*, ω), or in constant *Q* spectra by grouping the signal from neighboring detectors. Additional normalization steps and data analysis were carried out directly on the S(*Q* = const, ω) spectra using Microcal OriginPro 8.6. These normalizations considered A) the total quantity of material present in the beam and B) the correct quantity of buffer to be subtracted. With respect to point B), we had to take into account the volume fraction (ϕ = 5%) occupied by the bacterial cells. For step A) we note that the scattered intensity I(2Θ, t), and therefore S(*Q*, ω), is proportional to the amount of material in the beam and is already weighted for the corresponding coherent/incoherent cross section. Each set of S(*Q* = const, ω) spectra generated by the same S(*Q*, ω) matrix was normalized to the integral of S(*Q*, ω) over the entire *Q* range and the quasielastic ω range. The resolution of the time-of-flight experiment excluded any phononic contribution from the empty can.

The spectra were analyzed using a built-in least squares algorithm that takes into account a central Gaussian feature (to account for the energy resolution) along with up to three Lorentzian functions. The elastic component was removed to reveal the quasi-elastic line broadening of interest for each dataset as a function of the scattering vector *Q*.

Following data acquisition and discrimination of the QENS signal from the pure elastic, Gaussian scattering component determined by the instrument resolution function we proceeded with construction of the isotopic contrasts and comparison between 0.1 and 200 MPa profiles, followed by data fitting using a hierarchy of models developed to describe the relaxation dynamics and diffusion processes^6^,^7^,^8^,^9^,^10^,^11^,^40^. For studies of the bulk aqueous buffer media (Hb, Db) and the intracellular medium (Im) the data were best fit using two Lorentzian lineshapes representing translational relaxation corresponding to diffusional processes, and a rotational relaxation component. The QENS results across the cell membrane were best fit using only a single Lorentzian contribution related to the transmembrane diffusion (Fig. 1; Table 1). Each Lorentzian lineshape is associated with a half width at half maximum (Γ in meV) that reveals itself as a measure of the relaxation time for an excitation with correlation length *Q*, once it is converted into inverse timescale units (s^−1^) *via* ΔE = ħω. The translational linewidth (Γ__T__) is analyzed as a function of *Q* using different models for the transport process^6^,^7^,^8^,^40^.

The following protocols were applied to establish S(*Q*, ω) for the different cell/buffer combinations:

(i) S(*Q*, ω) [bacterial cells in buffer] = S(*Q*, ω) [bacterial cells in buffer within the Al HP container] – S(*Q*, ω) [empty container]

where cells (Hc, Dc) and aqueous buffer media (Db, Hb) were hydrogenated (H) or perdeuterated (D) varieties. A similar subtraction procedure was used for the buffer solutions alone:

(ii) S(*Q*, ω) [buffer] = S(*Q*, ω) [buffer within the Al HP container] – S(*Q*, ω) [empty container]

We initially applied a correction for self absorption by the empty high P Al container but this was neglected in later treatments as it made no difference to the results.

(iii) S(*Q*, ω) [bacterial cells] = S(*Q*, ω) [bacterial cells in buffer] – α × S(*Q*, ω) [buffer]

Here the factor α = (1–ϕ) accounts for the macromolecular volume fraction (5%) occupied by the bacterial cells in solution. In contrast to macromolecules such as proteins, with a composition that can be determined readily by sequencing and other assay techniques, the exact molecular composition of the live cells is not known precisely. However, because the buffer does not produce in any intense peak in the elastic region, the elastic scattering from the cells gives a reliable estimate of the macromolecular concentration. We thus estimated ϕ and α by normalizing the elastic intensity of (Dc/Db) to that of (Dc/Hb) at the first diffraction maximum (1.8 Å^−1^), where pure coherent scattering was expected to occur.

### Dataset analyses

The relationship


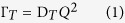


that introduces the diffusion coefficient D_T_ (cm^2^s^−1^) applies when interactions between molecules are weak, and in general as D_T_ → 0. Typically plots of Γ_T_ versus *Q*^*2*^ are analyzed to provide estimates of D_T_ that are generally found to be comparable with NMR and bulk transport measurements^6^,^7^,^8^,^40^.

As intramolecular interactions become significant the diffusion processes deviate from Fick’s law and the dynamics are described using two (or more) characteristic timescales. A typical approach involves the use of molecular jump models between sites separated by average length, l, and mean site residence time, τ_0_, during which the molecules undergo oscillatory motions:


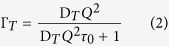



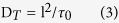


If the jump lengths are distributed randomly, the diffusion is usefully described by a Singwi-Sjölander (*SS*) jump model, in which each water molecule executes oscillations during a mean residence time (τ_0_) then diffuses during time τ_1_ to a second site^6^,^7^,^8^,^9^,^10^,^11^,^26^. Typically, 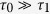 and the fitting model then reduces to:


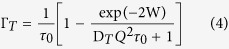


2W is a Debye-Waller factor 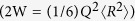 where 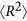 is the mean square displacement in the oscillatory part of the motion. Here we present our results using the *SS* model to analyze the QENS datasets, although other models were examined during the course of the study.

## Additional Information

**How to cite this article**: Foglia, F. *et al.* Water Dynamics in *Shewanella oneidensis* at Ambient and High Pressure using Quasi-Elastic Neutron Scattering. *Sci. Rep.* **6**, 18862; doi: 10.1038/srep18862 (2016).

## Figures and Tables

**Table 1:** Diffusion parameters extracted from fits by applying the *SS* jump model to fit Γ_T_(*Q* ^2^) within *Q* = 0.2–1.0 and 0.2–1.8 Å^−1^ ranges at 0.1 and 200 MPa.

Sample	Pressure/MPa	Diffusion parameters applying the *SS* jump model over Q-range indicated							τ_R_/ps	ΔP/%
		Q-range: 0.2–1.0 Å^−1^			Q-range: 0.2–1.8 Å^−1^					
		D_T_×10^−5^/cm^2^s^−1^	τ_0_/ps	2W	D_T_×10^−5^/cm^2^s^−1^	τ_0_/ps	2W	ΔP/%		
Hydrogenated buffer (Hb)	0.1	—	—	—	2.70 ± 0.20	1.0 ± 0.10	0.01	−11	1.68 ± 0.06	+ 18
	200	—	—	—	2.40 ± 0.19	1.22 ± 0.13	0.01		2.05 ± 0.09	
Deuterated buffer (Db)	0.1	—	—	—	2.00 ± 0.11	1.02 ± 0.09	0.02	−9	1.80 ± 0.06	+ 10
	200	—	—	—	1.82 ± 0.20	1.15 ± 0.16	0.02		2.00 ± 0.09	
Intracellular medium (Im) (Dc/Hb–Dc/Db)–Hb	0.1	—	—	—	2.43 ± 0.14	1.81 ± 0.07	0.05	−18	1.85 ± 0.10	+ 26
	200	—	—	—	2.00 ± 0.12	2.00 ± 0.10	0.05		2.50 ± 0.09	
[(Hc/Db)–Db]	0.1	2.12 ± 0.30	3.18 ± 0.21	0.17	2.57 ± 0.40	2.04 ± 0.10	0.07	−18	1.92 ± 0.12	+ 12
	200	1.67 ± 0.40	3.71 ± 0.19	0.24	2.11 ± 0.30	2.11 ± 0.09	0.08		2.18 ± 0.10	
[(Dc/Hb)–Hb]	0.1	2.12 ± 0.40	3.22 ± 0.26	0.17	2.45 ± 0.36	1.97 ± 0.25	0.07	−18	1.95 ± 0.09	+ 11
	200	1.82 ± 0.30	3.46 ± 0.22	0.20	2.05 ± 0.30	2.13 ± 0.08	0.08		2.20 ± 0.09	
[((H/D)–Im)–Db]	0.1	1.75 ± 0.36	2.27 ± 0.10	0.09	2.43 ± 0.24	1.89 ± 0.09	0.04	−20	—	
	200	1.52 ± 0.38	2.45 ± 0.33	0.09	1.95 ± 0.30	2.15 ± 0.08	0.06		—	
[((D/H)–Im)–Hb]	0.1	1.82 ± 0.25	2.21 ± 0.30	0.10	2.64 ± 0.36	2.01 ± 0.17	0.05	−21	—	
	200	1.52 ± 0.36	2.49 ± 0.27	0.11	2.10 ± 0.21	2.35 ± 0.12	0.05		—	

The rotational relaxational time (τ_R_) was extracted from the fit of the dynamic structure factor to the broad Lorentzian component.
